# Editorial: Safety and side effects of psychotropic medications, volume III

**DOI:** 10.3389/fpsyt.2025.1562008

**Published:** 2025-03-14

**Authors:** Renato de Filippis, Mireia Solerdelcoll, Mohammadreza Shalbafan

**Affiliations:** ^1^ Psychiatry Unit, Department of Health Sciences, University Magna Graecia of Catanzaro, Catanzaro, Italy; ^2^ Department of Child and Adolescent Psychiatry and Psychology, Institute of Neuroscience, Hospital Clínic de Barcelona, Barcelona, Spain; ^3^ Department of Medicine, University of Barcelona, Barcelona, Spain; ^4^ Mental Health Research Center, Psychosocial Health Research Institute (PHRI), Department of Psychiatry, School of Medicine, Iran University of Medical Sciences, Tehran, Iran; ^5^ Brain and Cognition Clinic, Institute for Cognitive Sciences Studies, Tehran, Iran

**Keywords:** adverse drug reaction (ADR), antidepressant, antipsychotic, mental disorders, mental health, psychotropic drugs, psychopharmacology, tolerability

Medications for mental disorders, like any other drug in any medical field, are burdened by side effects of varying severity and frequency, which characterize their safety and tolerability profile (1, 2). Indeed, while some ADRs are minor, other severe side effects can reduce quality of life, compromise medication adherence, lead to physical health problems, or, in rare cases, result in death (3–5). These risks must be carefully weighed against the therapeutic benefits of the medications, which are central to managing most major mental disorders (6–8). However, the stigma associated with psychiatry is particularly relevant in psychopharmacology, having the effect of tipping the scales mainly on the negative aspects, overshadowing the therapeutic advantages, especially noticeable in the case of clozapine (9, 10).

Completing the path of the first two volumes, this third chapter of the Research Topic entitled *Safety and Side Effects of Psychotropic Medications* collects eight different scientific contributions, diverse in their themes, target populations and results, but united by the centrality of evaluating the safety and tolerability of psychotropic therapies (11, 12).

In the paper by Zeiss et al., authors examined the relationship between receptor binding properties of antipsychotics and their hepatotoxic risks using data from the FDA Adverse Event Reporting System (FAERS), to identify signals for hepatic disorders, serving as a proxy for drug-induced liver injury (DILI). They concluded that significant DILI signals were observed for antipsychotics like chlorpromazine, loxapine, olanzapine, and quetiapine, with chlorpromazine and loxapine showing the highest relationship. A significant negative correlation was found between DILI risk and serotonin receptor 5-HT1A affinity, while a positive correlation was noted with cholinergic receptor affinity. These results suggest that serotonin and cholinergic systems may influence DILI risk from antipsychotics.

Kwaśny et al. carried out a systematic review of 5-Methoxy-N,N-dimethyltryptamine (5-MeO-DMT), a promising rapid-acting antidepressant, to assess the short-term safety and tolerability in clinical trials. Across three studies involving 78 participants, the review found a favorable short-term safety and tolerability profile, without serious ADRs or participant dropouts.

Zhang et al. conducted a case-control study to identify early risk factors and diagnostic indicators for effective pulmonary embolism (PE) management. This paper analyzed data from 10,077 inpatients with mental disorders at Shenzhen Kangning Hospital in 2020, identifying 65 PE cases and comparing 41 new PE cases with 41 matched controls based on age and gender. Authors identified a history of restraint, alcoholism, and the use of antipsychotics and benzodiazepines as significant predictors of PE in inpatients with mental disorders. The d-dimer test was shown to be an effective screening tool for PE in this population. These insights can aid clinicians in recognizing high-risk patients and implementing preventive measures to mitigate the occurrence of PE.

COVID-19 has also had an impact on the use of psychotropic drugs, and this aspect has been assessed by Yang et al., where authors evaluated changes in antipsychotic drug concentrations in hospitalized patients with mental disorders before and after COVID-19 infection, focusing on factors influencing these changes. The results indicated that antipsychotic concentrations increased post-COVID-19 infection in hospitalized patients with mental disorders, particularly for clozapine, aripiprazole, quetiapine, olanzapine, risperidone, and paliperidone, with clozapine showing the most pronounced rise. These changes are potentially due to the infection itself and interactions with traditional Chinese medicines or antibiotics. Monitoring and dose adjustments, particularly for clozapine, are crucial to manage these effects.

Komatsu et al. conducted a retrospective cross-sectional study about the relationship between hypnotic polypharmacy and the duration of hypnotic prescriptions using a large dataset from the Japan Medical Data Center. Among 112,256 adults prescribed hypnotics, 67.9% received hypnotic monotherapy, while 32.1% were prescribed hypnotic polypharmacy, with longer prescription durations significantly increasing the likelihood of polypharmacy. Initiating treatment with monotherapy and limiting prescription duration may help reduce the risk of polypharmacy and its associated risks.

The research group led by Takeshima et al. explored the prevalence and factors associated with benzodiazepine anxiolytic polypharmacy (BAP), which is discouraged by guidelines but commonly observed in practice. Researchers analyzed prescriptions for 104,796 adults prescribed benzodiazepine anxiolytics. BAP was defined as the concurrent use of two or more such medications, and was found in 12.6% of participants. In detail, the increased likelihood of BAP was associated with the use of hypnotic monotherapy (aOR: 1.04), antidepressant monotherapy (aOR: 1.57) and polypharmacy (aOR: 1.98), and antipsychotic monotherapy (aOR: 1.12) and polypharmacy (aOR: 1.41). The study concluded that patients requiring multiple psychotropic medications might exhibit resistance to standard pharmacological treatments, underscoring the need to emphasize non-pharmacological therapies to reduce BAP.

The study by Carmassi et al. evaluated the long-term real-world effectiveness and tolerability of cariprazine, a third-generation antipsychotic, in treating schizophrenia. Conducted over 12 months, it included 31 outpatients in Italy with DSM-5-TR schizophrenia diagnoses. According to their results, extrapyramidal symptoms were mild or mild-to-moderate, with no cases of moderate akathisia or dystonia. Finally, the study confirmed cariprazine’s efficacy in managing schizophrenia symptoms and its favorable tolerability regarding extrapyramidal side effects in a real-world, long-term setting.

Finally, the case report by Wiss et al. highlighted the challenges of interpreting therapeutic drug monitoring (TDM) and pharmacogenetic (PGx) testing in managing clozapine therapy. Authors reported the case of a 36-year-old male with catatonic schizophrenia who experienced recurrent hospitalizations due to elevated creatine kinase levels, suspected to be ADRs to clozapine. Despite low or subtherapeutic clozapine plasma levels during hospital admissions, no other medical cause was identified. This case highlights as isolated TDM or PGx results may lead to misinterpretation, while a comprehensive evaluation of patient history, adherence, dosing, and clinical context is essential. The report emphasizes cautious interpretation of TDM and PGx data, recommending a holistic approach to optimize clozapine therapy.

Although psychopharmacology is a rapidly expanding discipline that attracts great interest, much remains to be done to find the right balance between efficacy, tolerability and safety perceived by patients, healthcare professionals and caregivers (13, 14). Moreover, it would be desirable to increase collaborative care, including clinical pharmacists into the decision-making process, could further improve treatment outcomes and reduce medication-related issues. Indeed, transitions of care during hospital admission and discharge are crucial, and comprehensive management can help prevent medication problems (15, 16), particularly in addressing the management of polypharmacy, which significantly increases the risk of side effects, especially in vulnerable populations (17).

The articles collected in this, along with those from the preceding two volumes of this Research Topic, provide evidence-based insights into the management of side effects in psychopharmacology. While no drugs are without side effects, ongoing research continually strives to develop safer alternatives and more precise and tailored treatments for patients (18, 19).
